# Fatal Tracheostomy-Related Complications in a Pediatric Patient with DYT1 Dystonia After Delayed Deep Brain Stimulation

**DOI:** 10.7759/cureus.100892

**Published:** 2026-01-06

**Authors:** Cheng-En Wang, Chih-Fen Hu, Yuan-Hao Chen, Yueh-Feng Sung

**Affiliations:** 1 Department of Neurology, Tri-Service General Hospital, National Defense Medical University, Taipei, TWN; 2 Department of Pediatrics, Tri-Service General Hospital, National Defense Medical University, Taipei, TWN; 3 Department of Neurological Surgery, Tri-Service General Hospital, National Defense Medical University, Taipei, TWN

**Keywords:** airway compromise, clinical outcome, deep brain stimulation, dyt1, status dystonicus

## Abstract

The DYT1 dystonia is a rare autosomal dominant disorder characterized by early-onset focal dystonia, which may progress to generalized dystonia. Deep brain stimulation (DBS) is often effective in severe or medically refractory cases. We report a pediatric patient with a DYT1 mutation who developed focal dystonia at age six, which progressed to generalized dystonia by age eight, and culminated in status dystonicus at age 12. After stabilization, he remained in a state of refractory, generalized dystonia and underwent DBS following a seven-month delay. Although motor symptoms improved by 47%, he developed progressive airway dysfunction necessitating tracheostomy, which ultimately led to fatal respiratory complications. This case highlights that delayed DBS intervention may fail to prevent irreversible structural and neuromuscular sequelae, including fatal airway compromise, in patients with longstanding dystonia.

## Introduction

The DYT1 dystonia, caused by an in-frame GAG deletion in the TOR1A gene, is a rare autosomal dominant disorder with reduced penetrance [1]. Symptoms typically begin in one limb during childhood and may progress to involve the contralateral limb or trunk. Treatments include anticholinergics, benzodiazepines, baclofen, and botulinum toxin injections. When pharmacological treatment fails or when status dystonicus (also known as dystonic storm), a severe form of dystonia, develops, deep brain stimulation (DBS) has been shown to offer significant clinical benefit [2,3]. Although there are no established guidelines for the optimal timing of DBS, surgical intervention is generally considered before the development of irreversible musculoskeletal deformities or respiratory compromise.

We present the case of a pediatric patient with medically refractory DYT1 dystonia who underwent DBS seven months after an initial episode of status dystonicus. Although his dystonic symptoms improved postoperatively, he developed persistent airway compromise related to pre-existing cervical dystonia and prolonged ventilatory support, ultimately requiring tracheostomy. This case underscores the importance of timely surgical intervention in patients with severe dystonia and highlights the need for vigilant postoperative monitoring of airway function.

This work was previously presented as a poster at the International Congress of Parkinson’s Disease and Movement Disorders (MDS), held in Hawaii, USA, in October 2025.

## Case presentation

**Figure 1:**
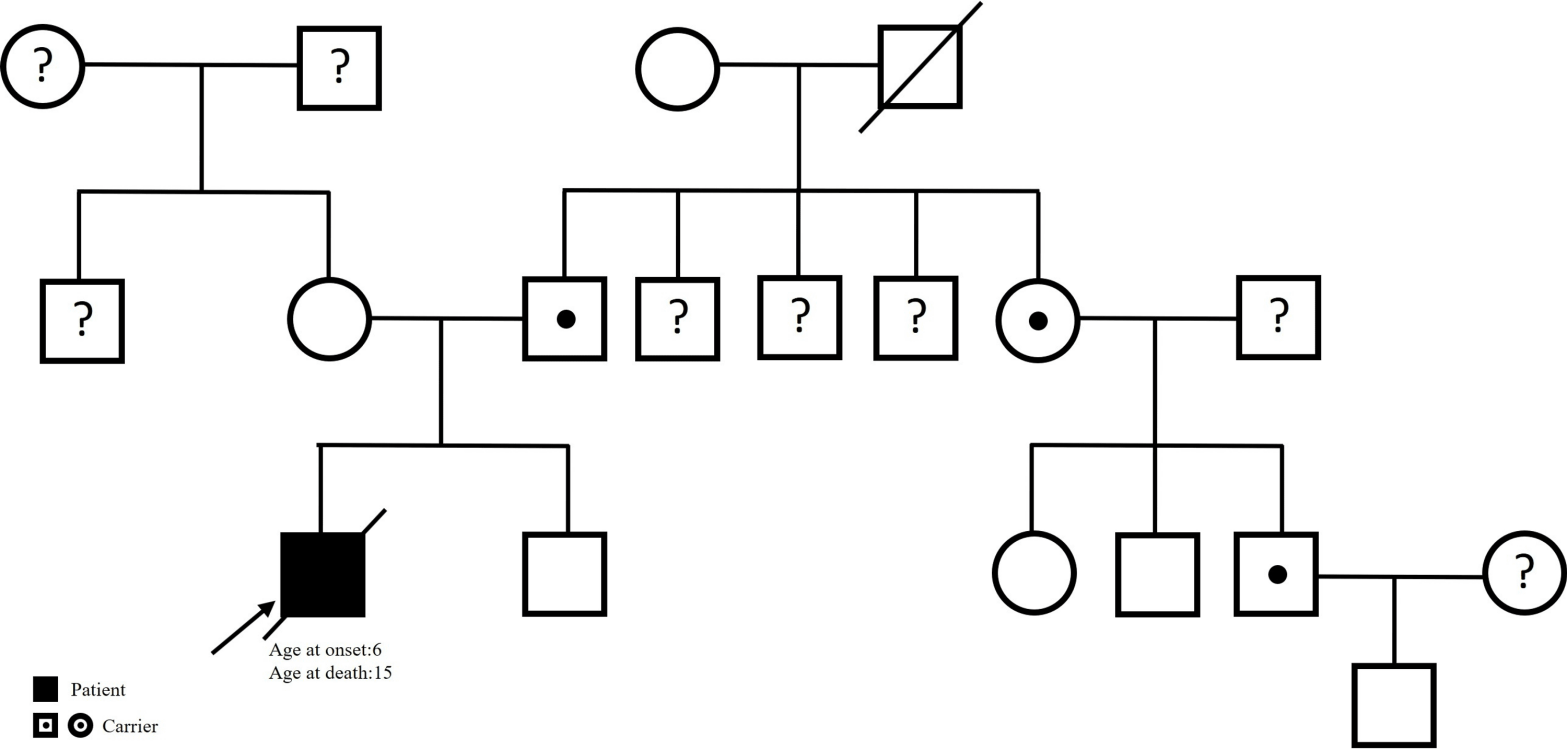
Family pedigree of the patient The index case is indicated by an arrow. The patient's father, aunt, and cousin are asymptomatic carriers (black dot). The question marks indicate the unknown status because we do not have DNA samples for study.

A previously healthy boy first developed right-leg twisting and gait disturbance at six years of age. Over the following two years, his dystonic symptoms gradually progressed to his upper limbs, trunk, and neck, accompanied by dystonic tremors that began in the right limbs and later extended to the left. All the symptoms disappeared during sleep. At eight years of age, when he presented to our neurology clinic, his height (121 cm) and weight (25 kg) were within the 15th-50th percentile range. Neurological examination revealed generalized dystonia involving the neck, trunk, and all four limbs, with preserved cognition. Genetic testing confirmed a pathogenic TOR1A mutation (exon 5: c.907_909delGAG, p.E303del). His father, aunt, and one cousin were found to be asymptomatic carriers (Figure 1).

Pharmacological treatments, including baclofen, clonazepam, and benzhexol hydrochloride, were initiated but discontinued due to adverse effects. Local botulinum toxin injections to the neck and limbs provided only partial relief. At age nine, the patient underwent bilateral femoral epiphysiodesis due to developmental dysplasia. His dystonia progressively worsened over the following year, rendering him bedridden. Although DBS was recommended, the family declined surgical intervention at the time. 

At age 12, he presented to the emergency department with high fever, dystonic opisthotonus, dysphagia, and anarthria. He was admitted to the intensive care unit with status dystonicus complicated by rhabdomyolysis, likely triggered by a urinary tract infection. His critical condition improved with supportive care, antibiotics, chloral hydrate, and continuous midazolam infusion. However, after resolution of the acute dystonic storm, he remained in a state of severe, medication-resistant generalized dystonia. He stayed in the prone position throughout the day, which appeared to function as a sensory trick to attenuate opisthotonus and maintain adequate oxygen saturation. Seven months later, the family consented to bilateral globus pallidus internus (GPi)-DBS, using Medtronic Model 3389 (Medtronic, Minneapolis, MN, USA) leads and an Activa RC Neurostimulator (Medtronic). Postoperatively, voltage-controlled monopolar stimulation resulted in substantial improvement. The Burke-Fahn-Marsden dystonia rating scale (BFMDRS) score improved from 112 preoperatively to 64 at two months (43% improvement) and 59 at 11 months (47% improvement) after surgery (Table 1) [4].

**Table 1:** BFMDRS scores before and after surgery The maximum BFMDRS score is 120; higher scores indicate greater severity of dystonia. BFMDRS: Burke-Fahn-Marsden dystonia rating scale

Variable	Time		
	Baseline	Two months	11 months
Eyes	8	0	0
Mouth	8	0	0
Speech and swallow	8	6	0
Neck	8	4	8
Right arm	16	9	9
Left arm	16	12	12
Right leg	16	12	6
Left leg	16	12	12
Trunk	16	9	12
Total	112	64	59

**Figure 2:**
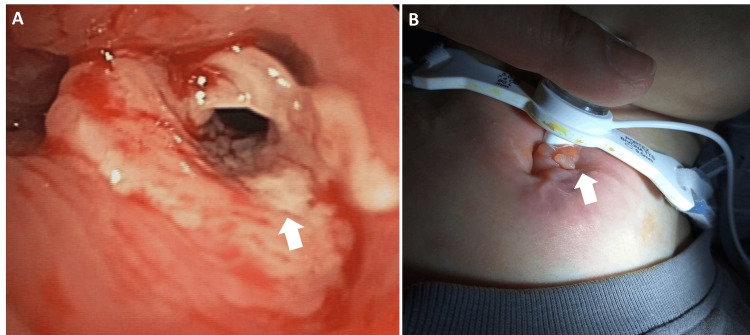
Trachea complication associated with long-term dystonia A: Granulation tissue (white arrow) within the trachea with erosive mucosal changes; B: Granulation tissue formation (white arrow) around the tracheostoma

Despite marked motor improvement, the patient continued to experience compromised airway function. Preoperatively, he exhibited intermittent, severe retrocollis, anterocollis, and torticollis. Following DBS, retrocollis and torticollis markedly improved, while anterocollis persisted as the predominant cervical posture. Intermittent truncal extension also remained. Three extubation attempts failed due to immediate upper airway obstruction, suspected to be related to tracheal collapse. During this period, he was not given neuromuscular blockade and was maintained on minimal ventilator support with low-pressure settings. Tracheostomy was ultimately performed 1.5 months after DBS, and he was subsequently weaned from mechanical ventilation. During hospitalization, the patient received bedside rehabilitation, including passive range-of-motion exercises and respiratory physiotherapy. He was discharged home six months postoperatively but experienced multiple readmissions due to recurrent pneumonia caused by methicillin-sensitive Staphylococcus aureus. Endoscopic evaluation revealed granulation tissue partially obstructing the airway, which was surgically removed. Nevertheless, he developed progressive tracheomalacia by age 14 (Figure 2).

Stimulation parameters were titrated gradually based on clinical response and tolerability. The most recent programming parameters 20 months post-DBS are listed in Table 2. No stimulation-induced pyramidal symptoms were observed during programming sessions. Unfortunately, due to the COVID-19 pandemic, the patient did not return for further DBS programming adjustments or airway evaluations. At age 15, he experienced acute bleeding from the tracheostomy site during the night, leading to complete airway obstruction. He was transported to the emergency department but was pronounced dead after unsuccessful resuscitation.

**Table 2:** Programming parameters 20 months after DBS DBS: Deep brain stimulation

Side	Contacts	Amplitude	Pulse width	Frequency
Left	0-C+ and 1-C+	6 V	80 μs	130 Hz
Right	8-C+, 9-C+, and 10-C+	9 V	80 μs	130 Hz

## Discussion

The DYT1 dystonia, caused by a GAG deletion in the TOR1A gene, is a rare autosomal dominant disorder and an uncommon cause of early-onset dystonia in Asian populations [1,5]. The classical phenotype involves childhood-onset focal dystonia, beginning in a lower limb, and potentially progressing to generalized dystonia or status dystonicus that can result in respiratory failure, rhabdomyolysis, and metabolic complications [2].

Deep brain stimulation is used in patients with medication-refractory dystonia or status dystonicus. A recent meta-analysis demonstrated that patients with TOR1A mutations showed a 69% improvement in motor symptoms after pallidal DBS, with poorer outcomes associated with longer disease duration in DYT1 dystonia [6]. A study by Tsuboi et al. in 2020 proposed that patients with more aggressive phenotypes, defined by early onset (five to 10 years), rapid progression within 2.5 years, and cranial involvement, tend to respond less favorably to DBS [7]. Although the optimal timing for surgery remains debated, emerging evidence from multicenter cohorts of monogenic dystonia suggests that irreversible musculoskeletal changes may negatively impact functional outcomes following pallidal DBS, reinforcing the importance of timely surgical consideration before the establishment of fixed deformities [2,8].

The effects of DBS on dystonia are time-dependent: phasic dystonic movements may resolve within minutes, while tonic posturing may require hours to days to improve [9]. In our case, generalized dystonia developed two years after disease onset, and DBS was performed six years after onset and seven months after the diagnosis of status dystonicus. An additional two months were required to optimize stimulation parameters, similar to adjustment periods reported in other series [10]. Notably, our patient exhibited all of these poor prognostic features [6,7], yet still achieved a 47% improvement on the BFMDRS one year postoperatively, a degree of improvement that is clinically relevant given the advanced disease stage and prolonged disease duration at the time of surgery.

Despite this marked motor improvement after DBS, the patient developed persistent airway compromise. Several factors likely contributed to the extubation failure and suspected tracheal collapse observed postoperatively. Chronic cervical dystonia may have caused abnormal spinal curvature, narrowing of the upper mediastinum, and patterned muscular contractions impairing airway patency [11]. Furthermore, his underweight status for age (body weight around 30 kg at 13 years) may have reflected immature or weakened tracheal cartilage, increasing susceptibility to dynamic airway collapse. During repeated extubation attempts, the patient exhibited anticipatory anxiety, which may have transiently exacerbated cervical dystonic tone. Such emotional arousal is known to trigger transient increases in dystonic activity, potentially destabilizing the airway in patients with pre-existing musculoskeletal vulnerabilities [12]. These factors, compounded by the underlying neuromuscular disorder, likely created a fragile airway environment prone to collapse following extubation.

The patient subsequently underwent tracheostomy due to failure of extubation attempts. Tracheomalacia later developed, likely as a late complication of prolonged tracheostomy tube placement. Chronic mechanical irritation, coupled with recurrent respiratory infections, may have contributed to progressive weakening of tracheal support structures, predisposing to dynamic airway collapse. This mechanism is well-recognized in children with prolonged tracheostomies, particularly those with neuromuscular disorders and low body weight [13]. These observations support the notion that post-tracheostomy care factors, superimposed on predisposing anatomical and neuromuscular vulnerabilities, likely contributed synergistically to the airway complications observed in our patient.

Postoperatively, while the patient showed marked improvement in oromandibular dystonia and was able to phonate using a speaking valve and take oral nutrition, residual dystonic posturing persisted. Dynamically, as indicated in Table 1, the BFMDRS neck subscore regressed to baseline severity (score of 8) at the 11-month follow-up. This regression suggests that while overall dystonia improved, cervical control remained refractory to stimulation, posing continued mechanical stress at the site of the tracheostomy. Accurately assessing the full extent of neck dystonia during routine outpatient visits is challenging. Even with DBS, patients may experience transient or phasic dystonic movements that, while no longer functionally disabling or affecting daily quality of life, still exert chronic, repetitive shearing forces against the tracheostomy-airway interface.

The patient later developed multiple tracheostomy-related complications, including granulation tissue, tracheomalacia, and ultimately fatal bleeding. Although post-mortem or angiographic confirmation was not available, the combination of persistent cervical dystonia with postoperative anterocollis and the sudden onset of massive hemorrhage from the tracheostomy site raises concern for a potential tracheo-innominate artery fistula (TIAF) as a contributing factor in the fatal outcome. A TIAF is a rare but well-documented and often fatal complication of long-term tracheostomy, particularly in pediatric patients with neurologic or musculoskeletal comorbidities, who may have abnormal cervical posture that increases the risk of anterior tracheal wall injury [14]. This case underscores the need for heightened clinical awareness of TIAF risk in patients with dystonia undergoing tracheostomy. In selected high-risk individuals, vascular surveillance such as CT angiography may be considered on a case-by-case basis.

## Conclusions

Early globus pallidus interna (GPi)-DBS should be strongly considered in cases of severe, medically refractory DYT1 dystonia to prevent complications associated with prolonged dystonia. In this case, although significant motor improvement was achieved following DBS, persistent airway compromise ensued, likely due to irreversible musculoskeletal changes and secondary tracheostomy-related pathology, rather than active dystonic processes alone. This case highlights the importance of comprehensive postoperative airway management in patients with severe dystonia undergoing tracheostomy. Such strategies may include minimizing airway irritation, preventing tracheal inflammation and granulation tissue formation, and considering routine bronchoscopy for early detection of complications.
